# Emodin in cardiovascular disease: The role and therapeutic potential

**DOI:** 10.3389/fphar.2022.1070567

**Published:** 2022-12-23

**Authors:** Yuanyuan Guo, Rongzhen Zhang, Wenlan Li

**Affiliations:** ^1^ School of Pharmacy, Harbin University of Commerce, Harbin, China; ^2^ Department of Cardiology, Geriatrics, and General Medicine, The First Affiliated Hospital of Harbin Medical University, Harbin, China; ^3^ Department of Heart Failure, Shanghai East Hospital, School of Medicine, Tongji University, Shanghai, China

**Keywords:** emodin, cardiovascular disease, pharmacological, effect, limitation

## Abstract

Emodin is a natural anthraquinone derivative extracted from Chinese herbs, such as *Rheum palmatum L*, *Polygonum cuspidatum*, and *Polygonum multiflorum*. It is now also a commonly used clinical drug and is listed in the Chinese Pharmacopoeia. Emodin has a wide range of pharmacological properties, including anticancer, antiinflammatory, antioxidant, and antibacterial effects. Many *in vivo* and *in vitro* experiments have demonstrated that emodin has potent anticardiovascular activity. Emodin exerts different mechanisms of action in different types of cardiovascular diseases, including its involvement in pathological processes, such as inflammatory response, apoptosis, cardiac hypertrophy, myocardial fibrosis, oxidative damage, and smooth muscle cell proliferation. Therefore, emodin can be used as a therapeutic drug against cardiovascular disease and has broad application prospects. This paper summarized the main pharmacological effects and related mechanisms of emodin in cardiovascular diseases in recent years and discussed the limitations of emodin in terms of extraction preparation, toxicity, and bioavailability-related pharmacokinetics in clinical applications.

## 1 Introduction

Cardiovascular disease (CVD), primarily ischemic heart disease and stroke, is the leading cause of death and disability worldwide (Roth et al., 2020; Tsao et al., 2022). CVD and mortality have been increasing globally since 1990, with the highest cardiovascular mortality in China. Hypertension is the most significant modifiable cardiovascular risk factor (Roth et al., 2020; China, 2022). Cardiovascular complications have been common in patients with coronavirus disease-2019 (COVID-19) pneumonia since the outbreak of the pandemic. Multiple studies have demonstrated that comorbid CVD is associated with a more severe course and higher mortality in COVID-19 pneumonia (Ma et al., 2021). In exploring its treatment, herbal remedies have made many positive contributions, demonstrating the unique efficacy of herbal medicine.

**FIGURE 1:**
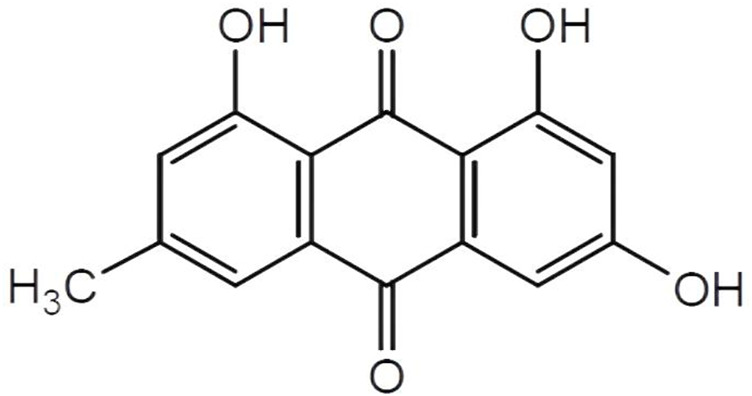
The structure of emodin. Emodin, 1,3,8-trihydroxy-6-methylanthracene-9,10-dione, molecular Formula is C_15_H_10_ O_5_. Emodin is a tricyclic planar structure with multiple modification sites, hydroxyl group at position 3, anthraquinone ring mainly at positions 2 and 4, and methyl group at position 6.

Chinese herbal medicines have been used in China for at least a 1000 years, and rhubarb is the collective name of a variety of perennial plants of the genus *Polygonaceae*. In China, rhubarb is primarily used for medicinal purposes. The Chinese herb rhubarb attacks stagnation, clears dampness and heat, dips fire, cools the blood, removes blood stasis, and detoxifies harmful toxins. Emodin, 1,3,8-trihydroxy-6-methylanthracene-9,10-dione (the structure of emodin is shown in Figure 1), is an anthraquinone compound extracted from rhubarb, with a molecular formula of C_15_H_10_O_5_. Emodin is a tricyclic planar structure with multiple modification sites: a hydroxyl group at position 3, an anthraquinone ring mainly at positions 2 and 4, and a methyl group at position 6 (Ghimire et al., 2015; Qiu et al., 2021). It is used abroad as a light laxative (Li et al., 2008). The pharmacokinetics of emodin show that emodin glucuronide/sulfate is present only in the plasma. Emodin is present in the kidney and lungs mainly as glucuronide/sulfate, and free emodin is present in high levels in the liver (Lin et al., 2012; Zhu et al., 2014). Wine processing increases the distribution of emodin in cardiopulmonary tissue (Wu et al., 2017). Polyethylene glycolytic drugs combined with emodin form stable emodin liposomes that can effectively increase emodin content in the heart (Wang et al., 2012). Laser confocal microscopy has shown that emodin is mostly dispersed in the cytoplasm and in small amounts in the nucleus (Wang et al., 2007). However, emodin has poor water solubility. Therefore, the modification of various points in its chemical structure could improve its water solubility appropriately, which is also a reasonable way to improve its bioavailability (Zheng et al., 2021). In this study, we integrated 18β-glycyrrhetinic acid and emodin, which not only improved emodin bioavailability but also reduced drug toxicity. This innovative one-step route has been shown to improve the hatching and survival rates of zebrafish embryos and reduce malformation and apoptosis rates of cardiomyocytes (Zhong et al., 2022). Emodin has shown many potential health benefits in preclinical models, with no significant toxicity to the cardiovascular system in rats, either through intraperitoneal or oral (20–80 mg/kg) administration (Sougiannis et al., 2021).

**FIGURE 2:**
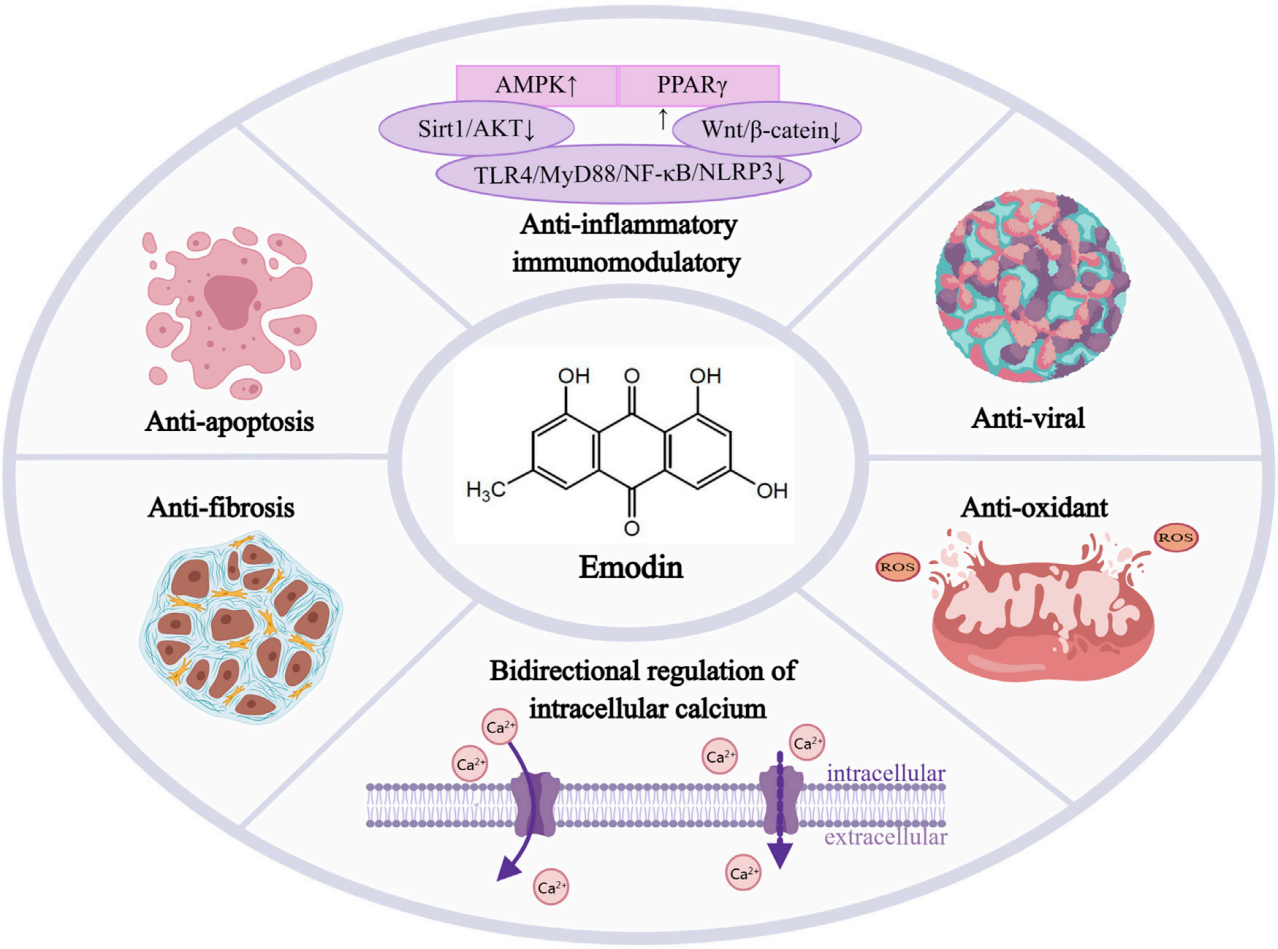
Emodin’s potent protective effects in cardiovascular diseases, Emodin has a series of protective effects in CVDs such as anti-inflammatory and immunomodulatory, anti-viral,anti-apoptosis, antioxidant and oxygen free radical scavenging, anti-fibrosis, and bidirectional regulation of intracellular calcium and L-type calcium electrodes in cardiac muscle. AMPK, activated protein kinase; PPARγ, peroxisome proliferator-activated receptor γ; Sirt1,sirtuin1; AKT, protein kinase B; TLR4,toll-like receptor-4; MyD88,myeloiddifferentiationfactor88; NF-κB, nuclear factor kappa-B; NLRP3,Nod-like receptors protein-3.

We first searched the National Library of Medicine website for the medical subject headings as subject terms “emodin” and “cardiovascular disease,a and then obtained the relevant professional subject terms and added them to the PubMed Search Builder to search the literature. It was found that emodin has cardioprotective activity (Tao et al., 2015) in atherosclerosis, myocardial ischemia-reperfusion (I/R) injury, myocardial hypertrophy, hypertension, and hyperlipidemia. The most recent results are shown in Table 1. In addition, multiple studies have confirmed that emodin has a series of protective effects in CVDs, such as antiinflammatory, immunomodulatory, antiviral, antioxidant and oxygen-free radical scavenging, antifibrosis, and bidirectional regulation of intracellular calcium and L-type calcium channels in cardiac muscle (as shown in Figure 2). This study summarized the effects of emodin on CVDs to provide a theoretical basis for clinical treatment.

**TABLE 1:** Role of emodin in cardiovascular diseases.

Disease	Bioactivity	Mechanism	References
Atherosclerosis	Anti-Inflammation	decrease the expression of MMP-2 and MMP-9 increase the expression of TIMP-1	Limin et al. (2018)
Myocardial infarction	Anti-apoptosis	up-regulation of miR-138, inactivated Sirt1/AKT and Wnt/β-catenin pathways. inhibit caspase-3 activation	Zhang et al. (2019), Wu et al. (2007)
	Anti-Inflammation	suppress the TLR4/MyD88/NF-κB/NLRP3 inflammasome pathway, inhibit gasdermin D-mediated pyroptosis. suppress TNF-α expression and NF-kκB activation	Ye et al. (2019), Wu et al. (2007)
	Anti-oxidant	enhancement in mitochondrial antioxidant components	Du and Ko (2006)
Heart failure	Anti-apoptosis	suppress complex I and p-ERK	Liu and Ning (2021)
	Anti-cardiac hypertrophy	inhibits histone deacetylase activity	Evans et al. (2020)
		activate SIRT3 signaling	Gao et al. (2020)
Hypertension	Suppression of tonic tension	inhibition of PKCδ	Lim et al. (2014)
	Anti-fibrosis	reduce TGF-β1,CTGF,MMP-2 and TIMP-2 expression	Chen et al. (2012a), Chen et al. (2014)
Valvular calcification	Anti-calcium accumulation	suppress AKT/FOXO1 signaling	Luo et al. (2022)
	Anti-proliferation	*via* the NF-κB signaling pathway by inhibiting the gene expression of BMP2, TNF, TRAF1, and RELA.	Xu et al. (2018b)
Viral myocarditis	Anti-virus	differentially regulate multiple signal cascades, including Akt/mTORC1/p70(S6K) (p70 S6 kinase), ERK1/2 (extracellular-signal-regulated kinase 1/2)/p90(RSK) (p90 ribosomal S6 kinase) and Ca(2+)/calmodulin	Zhang et al. (2016a)
	Anti-Inflammation	inhibiti IL-23/IL-17 inflammatory axis, Th17 cell proliferation and viral replication	Jiang et al. (2014)
	Anti-Inflammation	decrease the production of proinflammatory cytokines TNF-α and IL-1β	Song et al. (2012)
Septic myocardial injury	Anti-Inflammation	Inhibit NLRP3	Dai et al. (2021)
Diabetic cardiomyopathy	Anti-Inflammation	upregulation the AKT/GSK-3β Signaling Pathway	Wu et al. (2014)
Hyperlipidaemia	Anti-Inflammation	AMPK activation and PPARγ activation	Chen et al. (2012b), Tzeng et al. (2012)
		inhibits 11beta-hydroxysteroid dehydrogenase type 1	Feng et al. (2010)
	Calcium regulation	upregulation of L-type calcium channel expression	Zhao et al. (2009)

## 2 The role and mechanism of emodin in CVDs

### 2.1 Atherosclerosis

Coronary heart disease seriously affects human health, and its pathogenesis is closely related to atherosclerosis. The process of atherosclerosis includes endothelial cell injury, subendothelial lipid deposition, proliferation and migration of vascular smooth muscle cells (VSMCs), and monocyte adhesion, migration, and differentiation into macrophages. The therapeutic effect of emodin on coronary heart disease is closely associated with the process of atherosclerosis (Li et al., 2021). In addition, emodin can be used as an acoustic sensitizer for sonodynamic therapy as a potential approach for atherosclerosis treatment by exerting sonodynamic effects on THP-1 macrophages, inducing increased reactive oxygen species (ROS) production, cytoskeletal filament disruption, and apoptosis in THP-1-derived macrophages (Gao et al., 2011; Qian and Gao, 2018). *In vivo* experiments have also demonstrated that emodin-mediated sonodynamic therapy can significantly reduce the size of atherosclerotic plaques by decreasing the inflammatory response in them. At the same time, the expression of matrix metalloproteinase (MMP)-2 and MMP-9 in atherosclerotic plaques in mice was decreased, and the expression of TIMP-1 was increased, suggesting that emodin-mediated sonodynamic therapy can, to some extent, change the histological composition of plaques and exert a stabilizing effect on them (Limin et al., 2018). In a zebrafish model established on a high-fat diet, emodin treatment was effective in reducing lipid accumulation in blood vessels and liver and inhibiting the inflammatory response of vascular neutrophils. The specific mechanism was found to enhance low-density lipoprotein uptake, reverse cholesterol transport, and inhibit cholesterol synthesis (Wang et al., 2016; He et al., 2022). The lipid-lowering effect of emodin is similar to that of simvastatin, and additionally, it also restores aortic endothelial function and improves antioxidant capacity (Wu et al., 2021). Emodin induces apoptosis in VSMCs and exerts antismooth muscle cell proliferation effects. It also increases the production of ROS in cells and upregulates the level of p53 protein in a dose-dependent manner and has therapeutic potential for progressive arterial restenosis caused by abnormal proliferation and migration of vascular smooth muscle (Wang et al., 2007). Thus, emodin exerts antiatherosclerotic effects by exerting lipid-modulating, endothelial-protective, and antiinflammatory effects to attenuate and stabilize atherosclerotic plaques.

### 2.2 Myocardial infarction

Acute myocardial infarction (AMI) is characterized by an intense inflammatory response and increased apoptosis. Apoptosis is a mechanism that removes debris during tissue injury, and the amount of apoptosis after myocardial infarction is positively correlated with the extent of myocardial infarction. The degree of inflammatory response is also a major determinant of cardiac remodeling and function. *In vivo* experiments showed that emodin significantly increased the levels of cTnI and PGC-1 (specific markers directly reflecting cardiomyocyte energy metabolism) as well as the expression of complex I and p-ERK in the myocardial tissue from myocardial infarction mice. It also significantly improved myocardial energy metabolism, reduced myocardial apoptosis, and improved cardiac function in a dose-dependent manner (Liu and Ning, 2021). Emodin prevents myocardial cell injury by inhibiting local inflammatory responses and apoptosis. Administration of emodin reduced myocardial infarct size in a dose-dependent manner in mice with AMI, significantly inhibited tumor necrosis factor (TNF)-α expression and NF-κB activation in localized myocardial infarct areas, and inhibited cardiomyocyte apoptosis by inhibiting caspase-3 activation (Wu et al., 2007).

Reactive oxygen and calcium overload are involved in the mechanism of I/R injury, and emodin not only attenuates I/R injury in the heart, brain, kidney, and small intestine of rats and mice through antiinflammatory effects but also has antioxidant and calcium-modulating effects (Wang et al., 2022). After myocardial I/R injury, the expression level of gasdermin D-N domains is elevated in myocardial tissue, and emodin treatment inhibits the onset of cellular pyroptosis, attenuates the release of inflammatory mediators, and reduces the expression of gasdermin D-N domains by inhibiting the TLR4/MyD88/NF-κB/NLRP3 inflammatory vesicle pathway. These results suggest a new therapeutic target for emodin to improve myocardial I/R injury (Ye et al., 2019). In Sprague–Dawley rats administered with emodin, MDA production in the heart is significantly reduced after I/R, left ventricular function is improved, and infarct size is reduced (Sato et al., 2000). Emodin exhibits powerful free radical scavenging activity. Similarly, emodin has myocardial protective effects in both male and female rat heart models of myocardial I/R by enhancing the mitochondrial antioxidant components (Du and Ko, 2005). Low-dose emodin pretreatment and ischemic preconditioning prevent myocardial I/R injury through similar but not identical biochemical mechanisms (Du and Ko, 2006). In conclusion, emodin can reduce the apoptosis rate in the myocardial tissue, improve cardiac function, and alleviate post-myocardial infarction heart failure by regulating apoptosis and the oxidative stress response in a myocardial infarction model. However, whether emodin affects disease progression through other mechanisms remains to be explored in future studies.

### 2.3 Hypertension

Hypertension is an important risk factor for CVDs and VSMCs, and their contraction mechanisms are closely related to the development of hypertension. It was demonstrated that emodin inhibited phenylephrine-induced, deendothelialized aortic vasoconstriction in a concentration- and time-dependent manner, and its diastolic mechanism was verified in primary VSMCs. Emodin was found to inhibit myosin light chain kinase activity, attenuate contraction, and suppress calcium by blocking PKC-δ sensitization, leading to relaxation of VSMCs and vasodilation (Lim et al., 2014). This finding could contribute to the development of emodin as a tonicity modulator for the treatment of hypertension.

In addition, renal hypertension accounts for approximately 1–5% of the total number of patients with hypertension. Renal hypertensive rats were prepared by two-kidney one-clip surgeries and then treated with emodin, irbesartan, or their combination. The results showed that in the emodin treatment group, the left ventricular mass index, hydroxyproline, collagen content, MMP-2, and TIMP-2 expressions were significantly decreased; however, systolic blood pressure and angiotensin II (Ang II) content remained stable to some extent. The application of irbesartan alone or the combination of emodin and irbesartan significantly reduced systolic blood pressure and Ang II content. This finding suggests that emodin has no significant hypotensive effect on renal hypertension, but its combination with irbesartan inhibits ventricular fibrosis in Goldblatt hypertensive rats by reducing the expression of MMP-2 and TIMP-2 tissue inhibitors (Chen C. et al., 2012; Chen et al., 2014). Moreover, the combination of irbesartan and emodin provides better antifibrotic effects than single applications. Therefore, not only can emodin produce many beneficial effects on its own, but it can also be researched and developed as a compound drug in the future so that it can be used as an auxiliary drug ingredient to reduce the side-effects of other drugs and increase their efficacy.

### 2.4 Valvular heart disease

Calcific heart valve disease is a prevalent valve disease that usually occurs in older adults and is increasingly being recognized as a significant financial and health burden. In one study, human aortic valve interstitial cells were isolated and cultured from patients with calcific heart valve disease. The addition of TNF-α to the culture medium induced calcification in the cells, and the addition of emodin to the TNF-α conditioned medium did not cause severe toxicity to the cells. Rather, it was found to interfere with cell proliferation and inhibit the formation of calcific deposits in a dose-dependent manner. Treatment with emodin can reduce the serum levels of the proinflammatory cytokines, such as TNF-α and interleukin (IL)-1β, and inhibit TNF-α-induced calcification in human aortic valve interstitial cells *via* the NF-κB pathway (Xu et al., 2018b). Emodin also attenuates aortic valve calcification associated with high calcium, inhibits elevated calcium levels, and upregulates osteogenic genes and calcium accumulation in porcine aortic valve interstitial cells under high calcium conditions. p-AKT and p-FOXO1 were upregulated in porcine aortic valve interstitial cells under hypercalcemic conditions, and this upregulation could be reversed through emodin treatment. The inhibitory effect of emodin was reversed by the addition of AKT activator, suggesting that emodin alleviated hypercalcemia-associated valve calcification by inhibiting AKT/FOXO1 signaling (Luo et al., 2022). These findings provide new insights into the treatment strategies for clinical valve calcification.

### 2.5 Myocarditis

The inflammatory response is a double-edged sword, and current evidence shows that it has become a key point in regulating the pathogenesis and progression of CVDs. Emodin inhibits NF-κB levels in the myocardial tissue of rats with autoimmune myocarditis, improves left heart function, and reduces the severity of myocarditis (Song et al., 2012). Lipopolysaccharide (LPS) stimulates the inflammatory response of cells, which can reduce the viability of H9c2 cells, downregulate the expression of cyclin D1 in cells, induce apoptosis, and increase the release of IL-1β, IL-6, and TNF-α. High concentrations of emodin (≥20 μM) can reduce H9c2 cell viability, while low doses (10 μM) of emodin can reduce LPS-induced cardiomyocyte inflammatory damage, mainly by downregulating miR-223 to inactivate the JNK signaling pathway. Overexpression of miR-223 can weaken the myocardial protective effect of emodin on H9c2 cells. Therefore, miR-223 is considered to be the target of emodin antiinflammatory therapy, providing *in vitro* evidence that emodin has the potential for clinical treatment of myocarditis (Yang et al., 2019). Similarly, emodin ameliorates LPS-induced myocardial injury and cardiac insufficiency by inhibiting NLRP3 inflammasome activation to attenuate inflammatory response and cardiomyocyte scorching (Dai et al., 2021). Thus, emodin exerts antiinflammatory and protective effects against myocardial injury.

Emodin has an anthraquinone ring structure and belongs to the anthraquinone group, which has been shown to have virucidal and antiviral activity against RNA, DNA, enveloped, non-enveloped, ph-dependent, and independent viruses, such as hepatitis viruses, herpes viruses, and influenza viruses (Parvez et al., 2019). Viral myocarditis is an excessive inflammation caused by viral myocardial infection. The main pathogen is the coxsackie group B virus, but the antiviral effect of emodin is not direct inactivation of the virus. Emodin prevents coxsackie virus B3 (CVB3) myocarditis by inhibiting the IL-23/IL-17 inflammatory axis, Th17 cell proliferation, and viral replication in mice, significantly reducing IL-23, IL-17, IL-6, and IL-1β expression levels in the myocardium and serum of CVB3-infected mice (Jiang et al., 2014). Studies have shown that emodin inhibits CVB3 replication by impairing the translation machinery and inhibiting viral translation elongation (Zhang H. M. et al., 2016). For CVB4 infection, *in vitro* experiments showed that emodin inhibited CVB4 entry and replication in a concentration- and time-dependent manner and inhibited CVB4 infection-induced apoptosis. *In vivo* studies have shown a dose-dependent increase in survival, body weight, and mean time to death in mice orally administered different doses of emodin as well as a significant decrease in myocardial virus titers and pathological scores/lesions (Liu et al., 2013). Emodin also has strong inhibitory activity against CVB5, and studies have shown that emodin reduced IFN-α mRNA expression but significantly enhanced TNF-α expression during the first 0–4 h after infection in a concentration- and time-dependent manner (Liu et al., 2015). Furthermore, in a study of 92 patients with viral myocarditis, control and observation groups were set up. In the observation group, rhubarb was administered orally in combination with the therapeutic drugs, and it was found that rhubarb treatment effectively reduced the serum levels of IL-23, IL-17, and sCD40L in patients with viral myocarditis and at the same time improved the myocardial enzymatic levels and cardiac function in patients. This confirmed the antiviral effect of rhubarb in patients with viral myocarditis (Wu and Yang, 2018). Emodin exerts antiviral effects by blocking the replication process of the virus. Future clinical applications still need more accurate extraction of the active ingredients in rhubarb to better exploit the effects of the emodin components and reduce the side-effects caused by other impurities.

### 2.6 Diabetic cardiomyopathy

Emodin exerts antidiabetic and lipid-regulating effects by upregulating the expression of L-type calcium channels in the pancreas and heart of hyperlipidemic diabetic rats (Zhao et al., 2009). Currently, the leading cause of death in people with diabetes is CVD, and this complication of diabetes and CVD is known as diabetic cardiomyopathy. Emodin induced a dose-dependent increase in plasma superoxide dismutase activity in dyslipidemia-diabetic rats (Zhao et al., 2009). In addition, emodin was able to induce increased phosphorylation of protein kinase B and glycogen synthase kinase-3β in the myocardium of type 2 diabetic rats and improve cardiac function (Wu et al., 2014). It was also found that emodin exerts an antiinsulin resistance effect by downregulating miR-20b and thus, upregulating SMAD7, which improves glucose metabolism (Xiao et al., 2019a). Thus, emodin may not only lower glucose and blood lipids but may also have great therapeutic potential in diabetic cardiomyopathy and metabolic syndrome.

### 2.7 Heart failure

Pathological myocardial hypertrophy is a prominent feature of cardiac remodeling. It is an initial compensatory response to increased ventricular wall tension, in which sustained myocardial hypertrophy leads to cardiac malformations and dysfunction, eventually leading to ventricular dilatation and heart failure. In recent years, cardiac function has been found to be closely associated with mitochondrial metabolism. Sirt3 is a major member of the mitochondrial sirtuin family, a key regulator of mitochondrial metabolism and function. Overall deficiency of Sirt3 exacerbates cardiac function and is associated with hyperacetylation of key enzymes in the cardiac tricarboxylic acid cycle and production of lactate and NADH (Xu et al., 2020). Emodin blocks agonist-induced and pressure overload-mediated myocardial hypertrophy (Evans et al., 2021) and plays an important role in alleviating myocardial hypertrophy by regulating the mitochondrial Sirt3 signaling pathway (Gao et al., 2020; Guo et al., 2020). Inhibition of histone deacetylase enzyme attenuates pathological cardiac hypertrophy and enhances myofibril relaxation *in vitro* and *in vivo* at the molecular level (Travers et al., 2021). Emodin blocks cardiomyocyte hypertrophy induced by phenylephrine or the intracellular agonist fipronil (Evans et al., 2020). *In vitro* experiments have demonstrated that emodin is a histone deacetylase inhibitor that decreases histone deacetylase activity in cardiomyocytes to increase histone acetylation. *In vivo* experiments have also demonstrated that emodin inhibits histone deacetylase activity in Ang II-exposed mouse hearts, reducing pathological myocardial hypertrophy and myocardial fibrosis (Evans et al., 2020). Therefore, antimyocardial hypertrophy is an important target of emodin for preventing the development of heart failure.

Myocardial fibrosis has been implicated as a causative factor for the eventual development of heart failure. The hyperactivation of cardiac fibroblasts, including increased proliferation, migration, and collagen synthesis, is a key step in cardiac fibrosis. Emodin attenuates aortic constriction model-induced cardiac fibrosis in mice, inhibits Ang II-stimulated cardiac fibroblast activation and migration capacity, and significantly attenuates the upregulation of α-SMA expression and collagen I synthesis in fibroblasts (Xiao et al., 2019b). Studies have shown that metastasis-associated protein 3(MTA3) expression is associated with cardiac fibrosis, and that MTA3 inhibits the proliferation and migration of cardiac fibroblasts (Qin et al., 2015). In a model of fibrosis treated with emodin, the expression of MTA3 was found to be significantly upregulated, which was confirmed by both gene knockdown and overexpression. Emodin inhibited the activation of cardiac fibroblasts by upregulating MTA3, thereby reducing cardiac fibrosis (Xiao et al., 2019b). In addition, the activation of TGF-β and its signaling pathway is a major factor leading to fibrosis in several organs (Khalil et al., 2017). Emodin attenuated TGF-β1-mediated activation and fibrosis in cardiac fibroblasts. Further, in a TGF-β1-induced cardiac fibroblast model, emodin inhibited the activation of Erk1/2 and attenuated the expression of phosphorylated smad2/3, while phosphorylated p38 increased in a dose-dependent manner (Carver et al., 2021). These studies found that emodin can act as an effective antifibrotic agent by modulating multiple signaling pathways, thus reinforcing the idea that traditional Chinese medicine may provide a novel agent for improving the clinical management of cardiac fibrosis.

Calcium plays a crucial role in the maintenance of intracellular and extracellular homeostatic networks. Calcium homeostasis is critical for myocardial synchronization in heart failure. Abnormal cardiac diastolic calcium homeostasis can lead to impaired myocardial diastolic function, and abnormal myocyte calcium homeostasis in heart failure with reduced ejection fraction is associated with t-tubule disruption (Frisk et al., 2021). Emodin has a bidirectional effect on intracellular calcium concentrations and L-type calcium currents in adult guinea pig cardiomyocytes. At rest, intracellular calcium concentration was not affected by emodin concentration, whereas with the addition of potassium chloride, different emodin concentrations had different effects on intracellular calcium concentration and L-type calcium current in guinea pig cardiomyocytes, with inhibition of respective current at low concentrations (100 μm). At high concentrations (1 mm), it increased intracellular calcium concentration and L-type calcium current (Liu et al., 2004). In addition, experiments in isolated perfused beating rabbit atria showed that emodin also increased atrial natriuretic peptide secretion in a concentration-dependent manner, while decreasing atrial pulse pressure and beat-to-beat output. Inhibition of L-type calcium channels with nifedipine attenuates emodin-induced changes in atrial natriuretic peptide secretion and atrial kinetics (Zhou et al., 2022). This shows that the protective effect of emodin against CVDs and impaired regulation of cardiovascular endocytosis may be related to intracellular calcium ion levels and L-type calcium channels in cardiac myocytes. Experiments have only been performed under normal physiological conditions, and further exploration in disease models is needed.

## 3 Limitations of emodin application

Controversy still exists regarding the effects of emodin on tissue and cell toxicity, proliferation, and apoptosis, as well as the process of emodin preparation in preclinical trials. This included the dose of application, mode of administration, absorption and distribution, and the side-effects of diarrhea. All this leads to difficulties in initiating clinical trials with emodin.

### 3.1 Extraction technology

Emodin is a natural anthraquinone derivative that is widely found in Chinese herbal medicines, such as *Rheum palmatum*, *Rhizoma tigrinum*, and *Polygonum officinale*. Therefore, it has a wide range of sources and is widely used in medicine, health, food, and dye stuffs. Traditional extraction methods, such as marinated extraction, heat reflux extraction, and Soxhlet extraction, are very time-consuming and labor-intensive owing to the solubility properties of emodin. Modern extraction techniques, such as microwave-assisted extraction, can have harmful effects on the environment and human health because of the solubility properties of emodin, although this method is more efficient, faster, and consumes less solvent (Sun and Liu, 2008). A previous study proposed an ultrasound-assisted extraction method using natural deep eutectic solvents consisting of lactic acid, glucose, and water (Wu et al., 2018). Based on this, another study developed a more effective, rapid, simple, and economical extraction method by converting deep eutectic solvents into protic ionic liquids and using a microwave-assisted extraction method instead of ultrasound-assisted extraction (Fan et al., 2019). The extraction and preparation of emodin is still under further innovation and development.

### 3.2 Bioavailability

Regarding the pharmacokinetics and metabolism of emodin, after the administration of 20 mg/kg emodin by gavage, it can be rapidly absorbed by the circulatory system with a half-life of 6.44 h. However, the oral absorption bioavailability is only 2.83–3.2%, and about 56% of emodin is not absorbed and is excreted out of the body through feces. The absorbed components can be rapidly metabolized to hydroxylated and glucuronidated metabolites, which are mainly distributed in the kidney (Song et al., 2022; Zhou et al., 2022). When rats were given intravenous emodin 0.4 mg/kg, it was rapidly metabolized and eliminated, with a half-life of 1.82 h (Song et al., 2022). To improve the oral utilization of emodin, a study was conducted to prepare emodin nanoemulsions enriched with polyoxyethylene castor oil (Zhang T. et al., 2016). Given the lipid solubility of emodin, some studies have also configured emodin-liposome coupling agents, in which D-α-tocopheryl polyethylene glycol 1,000 succinate and a pegylated agent improved the encapsulation efficiency and stability of emodin egg phosphatidylcholine/cholesterol liposomes. This also prolonged the residence time of emodin in the circulatory system and increased its content in the heart (Wang et al., 2012). In addition, it was found that the distribution of emodin in the heart and lung tissues of rats was significantly altered by wine processing—a traditional Chinese method of “wine processing” (Wu et al., 2017). The encapsulation of drugs by nanoparticles is beneficial for improving the targeting and bioavailability of drugs and reducing their toxic side-effects (Li et al., 2017). For emodin to play a more targeted role in the cardiovascular system, additional preparation processes are still under development, such as increasing the anticardiovascular activity of emodin by adding modifications and loading in combination with nano-targeting systems. A large number of experiments are required to validate this in the future to lay the foundation for clinical applications.

### 3.3 Toxicity

In a wide variety of disease models, the effects of different emodin concentrations are not the same, and according to previous experimental analyses, most of the different effects of emodin are concentration-dependent and act differently in different cells. Because the anthracycline ring in the structure of emodin is very similar to the core of anthracyclines used in cancer therapy (adriamycin, erythromycin, etc.), emodin has cytotoxic and growth-inhibitory effects on different types of cancer cells. Additionally, long-term high doses of emodin may cause nephrotoxicity, hepatotoxicity, and reproductive toxicity (Akkol et al., 2021). At low concentrations (less than 20 μm), emodin did not affect the viability of cardiomyocytes and protected them from damage in a hypoxic environment (Yang et al., 2019; Zhang et al., 2019). In contrast, at the same low concentration (5 μm), emodin significantly inhibited the proliferation of VSMCs and vascular endothelial cells. The inhibition of mitochondrial activity in VSMCs was more pronounced than that in vascular endothelial cells (Xu et al., 2018a). *In vitro* experiments have shown that when emodin affects cardiomyocyte viability at a concentration of 20 μm, it also inhibits fibroblast proliferation; at 10 μm, it protects cardiomyocytes from ischemic and hypoxic injury, and attenuates TG-β-induced fibroblast proliferation (Carver et al., 2021). Therefore, the therapeutic application of emodin requires careful evaluation of its effects on specific cell types and exploration of the appropriate and most beneficial therapeutic concentrations. In addition, a deeper study of the specific mechanisms of emodin in different cells and tissues is needed when targeting different diseases for treatment.

## 4 Conclusion

Emodin is a natural bioactive drug that exerts its anticardiovascular activity through different molecular targets in vascular cells. Emodin regulates the inflammatory response, suppresses apoptosis, alleviates hypertrophy, and reduces myocardial fibrosis. It has promising prospects in myocardial ischemia, atherosclerosis, viral myocarditis, hypertension, and heart failure. Although encouraging results have been achieved in this research field, most are still in the preclinical experimental stage. Notably, clinical data supporting the safety and pharmacokinetics of emodin in humans are lacking. Therefore, there may be undetermined drawbacks in the clinical use of emodin. In conclusion, the current study represents an important step in the clinical development of emodin as an active agent against CVD.

## Glossary

CVD: Cardiovascular disease
ROS: reactive oxygen species
THP-1: human acute monocytic leukemia cell
MMP: matrix metalloproteinase
TIMP: tissue inhibitor of metalloproteinase-1
cTnI: cardiac troponin I
PGC-1: peroxisome proliferator-activated receptor-γ coactivator-1
p-ERK: phosphorylated-extracellular signal-regulated kinase
JNK: Jun kinase enzyme
TNF-α: tumor necrosis factor-alpha
NF-κB: nuclear factor kappa-B
IR: Ischemia-Reperfusion
TLR4: toll-like receptor-4
MyD88: myeloiddifferentiationfactor88
NLRP3: Nod-like receptors protein-3
MDA: malondialdehyde
VSMCs: vascular smooth muscle cells
PKC: protein kinase C
Ang II: angiotensin II
IL-1b: interleukin-1b
IL-6: interleukin-6
IL-23: interleukin-23
IL-17: interleukin-17
p-AKT: phosphorylated-protein kinase B
p-FOXO1: forkhead box protein O1
LPS: Lipopolysaccharide
miR: micro RNA
Th17**CVB3**: **CVB3**coxsackievirus B3
CVB4: coxsackievirus B4
CVB5: coxsackievirus B5
IFN-α: interferon-alpha
TNF-γ: tumor necrosis factor-gama
SMAD7: small mother against decapentaplegic-7
Sirt3: sirtuin 3
NADH: reduced form of nicotinamide-adenine dinucleotid
α-SMA: alpha-smooth muscle actin
MTA3: metastasis-associated protein 3
TGF-β: transforming growth factor-beta

## References

[B1] Akkol E. K. Tatli, II Karatoprak G. S. Agar O. T. Yucel C. Sobarzo-Sanchez E. (2021). Is emodin with anticancer effects completely innocent? Two sides of the coin. Cancers (Basel) 13 (11), 2733. 10.3390/cancers13112733 34073059 PMC8198870

[B2] Carver W. Fix E. Fix C. Fan D. Chakrabarti M. Azhar M. (2021). Effects of emodin, a plant-derived anthraquinone, on TGF-β1-induced cardiac fibroblast activation and function. J. Cell Physiol. 236 (11), 7440–7449. 10.1002/jcp.30416 34041746 PMC8530838

[B3] Chen C. Liang Z. Chen Q. Li Z. G. (2012a). Irbesartan and emodin on myocardial remodeling in Goldblatt hypertensive rats. J. Cardiovasc Pharmacol. 60 (4), 375–380. 10.1097/FJC.0b013e3182650f1c 23064242

[B4] Chen Q. Pang L. Huang S. Lei W. Huang D. (2014). Effects of emodin and irbesartan on ventricular fibrosis in Goldblatt hypertensive rats. Pharmazie 69 (5), 374–378. 24855831

[B5] Chen Z. Zhang L. Yi J. Yang Z. Zhang Z. Li Z. (2012b). Promotion of adiponectin multimerization by emodin: A novel AMPK activator with pparγ-agonist activity. J. Cell Biochem. 113 (11), 3547–3558. 10.1002/jcb.24232 22730200

[B6] China (2022). China cardiovascular health and disease report 2021 summary. Chin. Circulation J. 37 (06), 553–578.

[B7] Dai S. Ye B. Chen L. Hong G. Zhao G. Lu Z. (2021). Emodin alleviates LPS-induced myocardial injury through inhibition of NLRP3 inflammasome activation. Phytother. Res. 35 (9), 5203–5213. 10.1002/ptr.7191 34131970

[B8] Du Y. Ko K. M. (2005). Effects of emodin treatment on mitochondrial ATP generation capacity and antioxidant components as well as susceptibility to ischemia-reperfusion injury in rat hearts: Single versus multiple doses and gender difference. Life Sci. 77 (22), 2770–2782. 10.1016/j.lfs.2005.03.027 15964600

[B9] Du Y. Ko K. M. (2006). Effects of pharmacological preconditioning by emodin/oleanolic acid treatment and/or ischemic preconditioning on mitochondrial antioxidant components as well as the susceptibility to ischemia-reperfusion injury in rat hearts. Mol. Cell Biochem. 288 (1-2), 135–142. 10.1007/s11010-006-9129-3 16583138

[B10] Evans L. Shen Y. Bender A. Burnett L. E. Li M. Habibian J. S. (2021). Divergent and overlapping roles for selected phytochemicals in the regulation of pathological cardiac hypertrophy. Molecules 26 (5), 1210. 10.3390/molecules26051210 33668293 PMC7956446

[B11] Evans L. W. Bender A. Burnett L. Godoy L. Shen Y. Staten D. (2020). Emodin and emodin-rich rhubarb inhibits histone deacetylase (HDAC) activity and cardiac myocyte hypertrophy. J. Nutr. Biochem. 79, 108339. 10.1016/j.jnutbio.2019.108339 32007664 PMC7162729

[B12] Fan Y. Niu Z. Xu C. Yang L. Yang T. (2019). Protic ionic liquids as efficient solvents in microwave-assisted extraction of rhein and emodin from Rheum palmatum L. Molecules 24 (15), 2770. 10.3390/molecules24152770 31366111 PMC6695579

[B13] Feng Y. Huang S. L. Dou W. Zhang S. Chen J. H. Shen Y. (2010). Emodin, a natural product, selectively inhibits 11beta-hydroxysteroid dehydrogenase type 1 and ameliorates metabolic disorder in diet-induced obese mice. Br. J. Pharmacol. 161 (1), 113–126. 10.1111/j.1476-5381.2010.00826.x 20718744 PMC2962821

[B14] Frisk M. Le C. Shen X. Roe A. T. Hou Y. Manfra O. (2021). Etiology-dependent impairment of diastolic cardiomyocyte calcium homeostasis in heart failure with preserved ejection fraction. J. Am. Coll. Cardiol. 77 (4), 405–419. 10.1016/j.jacc.2020.11.044 33509397 PMC7840890

[B15] Gao J. Zhang K. Wang Y. Guo R. Liu H. Jia C. (2020). A machine learning-driven study indicates emodin improves cardiac hypertrophy by modulation of mitochondrial SIRT3 signaling. Pharmacol. Res. 155, 104739. 10.1016/j.phrs.2020.104739 32135248

[B16] Gao Q. Wang F. Guo S. Li J. Zhu B. Cheng J. (2011). Sonodynamic effect of an anti-inflammatory agent--emodin on macrophages. Ultrasound Med. Biol. 37 (9), 1478–1485. 10.1016/j.ultrasmedbio.2011.05.846 21767904

[B17] Ghimire G. P. Koirala N. Pandey R. P. Jung H. J. Sohng J. K. (2015). Modification of emodin and aloe-emodin by glycosylation in engineered Escherihia coli. World J. Microbiol. Biotechnol. 31 (4), 611–619. 10.1007/s11274-015-1815-4 25663173

[B18] Guo R. Liu N. Liu H. Zhang J. Zhang H. Wang Y. (2020). High content screening identifies licoisoflavone A as a bioactive compound of Tongmaiyangxin Pills to restrain cardiomyocyte hypertrophy via activating Sirt3. Phytomedicine 68, 153171. 10.1016/j.phymed.2020.153171 32018211

[B19] He L. F. Wang C. Zhang Y. F. Guo C. C. Wan Y. Li Y. X. (2022). Effect of emodin on hyperlipidemia and hepatic lipid metabolism in zebrafish larvae fed a high-cholesterol diet. Chem. Biodivers. 19 (2), e202100675. 10.1002/cbdv.202100675 34866324

[B20] Jiang N. Liao W. Kuang X. (2014). Effects of emodin on IL-23/IL-17 inflammatory axis, Th17 cells and viral replication in mice with viral myocarditis. Nan Fang. Yi Ke Da Xue Xue Bao 34 (3), 373–378. 24670452

[B21] Khalil H. Kanisicak O. Prasad V. Correll R. N. Fu X. Schips T. (2017). Fibroblast-specific TGF-beta-Smad2/3 signaling underlies cardiac fibrosis. J. Clin. Invest. 127 (10), 3770–3783. 10.1172/JCI94753 28891814 PMC5617658

[B22] Li D. Liu L. Yang S. Xing Y. Pan L. Zhao R. (2021). Exploring the therapeutic mechanisms of huzhang-shanzha herb pair against coronary heart disease by network pharmacology and molecular docking. Evid. Based Complement. Altern. Med. 2021, 5569666. 10.1155/2021/5569666 PMC8651359 34887932

[B23] Li F. Wang S. C. Wang X. Ren Q. Y. Wang W. Shang G. W. (2008). Novel exploration of cathartic pharmacology induced by rhubarb. Zhongguo Zhong Yao Za Zhi 33 (4), 481–484. 18533509

[B24] Li L. Sheng X. Zhao S. Zou L. Han X. Gong Y. (2017). Nanoparticle-encapsulated emodin decreases diabetic neuropathic pain probably via a mechanism involving P2X3 receptor in the dorsal root ganglia. Purinergic Signal 13 (4), 559–568. 10.1007/s11302-017-9583-2 28840511 PMC5714846

[B25] Lim K. M. Kwon J. H. Kim K. Noh J. Y. Kang S. Park J. M. (2014). Emodin inhibits tonic tension through suppressing PKCδ-mediated inhibition of myosin phosphatase in rat isolated thoracic aorta. Br. J. Pharmacol. 171 (18), 4300–4310. 10.1111/bph.12804 24909118 PMC4241095

[B26] Limin W. youwang L. Enbo W. Zhaolong Y. Yalin L. (2018). Study on the effect of emodin as acoustic sensitizer mediating acoustic dynamic therapy on atherosclerosis in mice. Med. Inf. 31 (06), 58–60.

[B27] Lin S. P. Chu P. M. Tsai S. Y. Wu M. H. Hou Y. C. (2012). Pharmacokinetics and tissue distribution of resveratrol, emodin and their metabolites after intake of Polygonum cuspidatum in rats. J. Ethnopharmacol. 144 (3), 671–676. 10.1016/j.jep.2012.10.009 23069945

[B28] Liu J. Ning L. (2021). Protective role of emodin in rats with post-myocardial infarction heart failure and influence on extracellular signal-regulated kinase pathway. Bioengineered 12 (2), 10246–10253. 10.1080/21655979.2021.1983977 34839778 PMC8809930

[B29] Liu Z. Ma N. Zhong Y. Yang Z. Q. (2015). Antiviral effect of emodin from Rheum palmatum against coxsakievirus B5 and human respiratory syncytial virus *in vitro* . J. Huazhong Univ. Sci. Technol. Med. Sci. 35 (6), 916–922. 10.1007/s11596-015-1528-9 PMC7089517 26670446

[B30] Liu Z. Wei F. Chen L. J. Xiong H. R. Liu Y. Y. Luo F. (2013). *In vitro* and *in vivo* studies of the inhibitory effects of emodin isolated from Polygonum cuspidatum on Coxsakievirus B₄. Molecules 18 (10), 11842–11858. 10.3390/molecules181011842 24071990 PMC6269740

[B31] Luo M. Sun W. Bin Z. Kong X. (2022). Emodin alleviates aortic valvular calcification by inhibiting the AKT/FOXO1 pathway. Ann. Anat. 240, 151885. 10.1016/j.aanat.2021.151885 34958913

[B32] Ma L. Song K. Huang Y. (2021). Coronavirus disease-2019 (COVID-19) and cardiovascular complications. J. Cardiothorac. Vasc. Anesth. 35 (6), 1860–1865. 10.1053/j.jvca.2020.04.041 32451271 PMC7192093

[B33] Parvez M. K. Al-Dosari M. S. Alam P. Rehman M. Alajmi M. F. Alqahtani A. S. (2019). The anti-hepatitis B virus therapeutic potential of anthraquinones derived from Aloe vera. Phytother. Res. 33 (11), 2960–2970. 10.1002/ptr.6471 31410907

[B34] Qian J. Gao Q. (2018). Sonodynamic therapy mediated by emodin induces the oxidation of microtubules to facilitate the sonodynamic effect. Ultrasound Med. Biol. 44 (4), 853–860. 10.1016/j.ultrasmedbio.2017.12.016 29398130

[B35] Qin W. Du N. Zhang L. Wu X. Hu Y. Li X. (2015). Genistein alleviates pressure overload-induced cardiac dysfunction and interstitial fibrosis in mice. Br. J. Pharmacol. 172 (23), 5559–5572. 10.1111/bph.13002 25362897 PMC4667871

[B36] Qiu X. Pei H. Ni H. Su Z. Li Y. Yang Z. (2021). Design, synthesis and anti-inflammatory study of novel N-heterocyclic substituted Aloe-emodin derivatives. Chem. Biol. Drug Des. 97 (2), 358–371. 10.1111/cbdd.13788 32889741

[B37] Roth G. A. Mensah G. A. Johnson C. O. Addolorato G. Ammirati E. Baddour L. M. (2020). Global burden of cardiovascular diseases and risk factors, 1990-2019: Update from the GBD 2019 study. J. Am. Coll. Cardiol. 76 (25), 2982–3021. 10.1016/j.jacc.2020.11.010 33309175 PMC7755038

[B38] Sato M. Maulik G. Bagchi D. Das D. K. (2000). Myocardial protection by protykin, a novel extract of trans-resveratrol and emodin. Free Radic. Res. 32 (2), 135–144. 10.1080/10715760000300141 10653484

[B39] Song Y. Yang J. Wang X. Chen J. Si D. Gao H. (2022). Pharmacokinetics and metabolism of trans-emodin dianthrones in rats. J. Ethnopharmacol. 290, 115123. 10.1016/j.jep.2022.115123 35183691

[B40] Song Z. C. Wang Z. S. Bai J. H. Li Z. Hu J. (2012). Emodin, a naturally occurring anthraquinone, ameliorates experimental autoimmune myocarditis in rats. Tohoku J. Exp. Med. 227 (3), 225–230. 10.1620/tjem.227.225 22791134

[B41] Sougiannis A. T. Enos R. T. VanderVeen B. N. Velazquez K. T. Kelly B. McDonald S. (2021). Safety of natural anthraquinone emodin: An assessment in mice. BMC Pharmacol. Toxicol. 22 (1), 9. 10.1186/s40360-021-00474-1 33509280 PMC7845031

[B42] Sun C. Liu H. (2008). Application of non-ionic surfactant in the microwave-assisted extraction of alkaloids from Rhizoma Coptidis. Anal. Chim. Acta 612 (2), 160–164. 10.1016/j.aca.2008.02.040 18358861

[B43] Tao S. Liang X. Y. Wang Y. Wang Y. (2015). Screening of active compounds with myocardial protective effects from Tongmai Yangxin pill. Zhejiang Da Xue Xue Bao Yi Xue Ban. 44 (2), 145–153. 10.3785/j.issn.1008-9292.2015.03.005 26038132 PMC10396997

[B44] Travers J. G. Wennersten S. A. Pena B. Bagchi R. A. Smith H. E. Hirsch R. A. (2021). HDAC inhibition reverses preexisting diastolic dysfunction and blocks covert extracellular matrix remodeling. Circulation 143 (19), 1874–1890. 10.1161/CIRCULATIONAHA.120.046462 33682427 PMC8884170

[B45] Tsao C. W. Aday A. W. Almarzooq Z. I. Alonso A. Beaton A. Z. Bittencourt M. S. (2022). Heart disease and stroke statistics-2022 update: A report from the American heart association. Circulation 145 (8), e153–e639. 10.1161/CIR.0000000000001052 35078371

[B46] Tzeng T. F. Lu H. J. Liou S. S. Chang C. J. Liu I. M. (2012). Emodin, a naturally occurring anthraquinone derivative, ameliorates dyslipidemia by activating AMP-activated protein kinase in high-fat-diet-fed rats. Evid. Based Complement. Altern. Med. 2012, 781812. 10.1155/2012/781812 PMC3357974 22649478

[B47] Wang J. Ji J. Song Z. Zhang W. He X. Li F. (2016). Hypocholesterolemic effect of emodin by simultaneous determination of *in vitro* and *in vivo* bile salts binding. Fitoterapia 110, 116–122. 10.1016/j.fitote.2016.03.007 26964768

[B48] Wang T. Yin X. Lu Y. Shan W. Xiong S. (2012). Formulation, antileukemia mechanism, pharmacokinetics, and biodistribution of a novel liposomal emodin. Int. J. Nanomedicine 7, 2325–2337. 10.2147/ijn.S31029 22661889 PMC3357979

[B49] Wang X. Zou Y. Sun A. Xu D. Niu Y. Wang S. (2007). Emodin induces growth arrest and death of human vascular smooth muscle cells through reactive oxygen species and p53. J. Cardiovasc Pharmacol. 49 (5), 253–260. 10.1097/FJC.0b013e318033dfb3 17513942

[B50] Wang Y. Liu Q. Cai J. Wu P. Wang D. Shi Y. (2022). Emodin prevents renal ischemia-reperfusion injury via suppression of CAMKII/DRP1-mediated mitochondrial fission. Eur. J. Pharmacol. 916, 174603. 10.1016/j.ejphar.2021.174603 34793771

[B51] Wu c. Yang H. (2018). Effect of rhodopsin on the efficacy and serum indices of patients with viral myocarditis. Clin. Medicat. J. 16 (09), 39–42+47.

[B52] Wu J. H. Lv C. F. Guo X. J. Zhang H. Zhang J. Xu Y. (2021). Low dose of emodin inhibits hypercholesterolemia in a rat model of high cholesterol. Med. Sci. Monit. 27, e929346. 10.12659/MSM.929346 34257265 PMC8287934

[B53] Wu Y. C. Wu P. Li Y. B. Liu T. C. Zhang L. Zhou Y. H. (2018). Natural deep eutectic solvents as new green solvents to extract anthraquinones from Rheum palmatum L. RSC Adv. 8 (27), 15069–15077. 10.1039/c7ra13581e 35541349 PMC9079993

[B54] Wu Y. Peng X. Q. Jiang X. Y. Shi M. Q. Yang S. Y. Fu Y. J. (2017). Effects of wine processed Rheum palmatum on tissue distribution of aloe-emodin, rhein and emodin in rats. Zhongguo Zhong Yao Za Zhi 42 (8), 1603–1608. 10.19540/j.cnki.cjcmm.20170224.014 29071869

[B55] Wu Y. Tu X. Lin G. Xia H. Huang H. Wan J. (2007). Emodin-mediated protection from acute myocardial infarction via inhibition of inflammation and apoptosis in local ischemic myocardium. Life Sci. 81 (17-18), 1332–1338. 10.1016/j.lfs.2007.08.040 17939930

[B56] Wu Z. Chen Q. Ke D. Li G. Deng W. (2014). Emodin protects against diabetic cardiomyopathy by regulating the AKT/GSK-3β signaling pathway in the rat model. Molecules 19 (9), 14782–14793. 10.3390/molecules190914782 25232702 PMC6271268

[B57] Xiao D. Hu Y. Fu Y. Wang R. Zhang H. Li M. (2019a). Emodin improves glucose metabolism by targeting microRNA-20b in insulin-resistant skeletal muscle. Phytomedicine 59, 152758. 10.1016/j.phymed.2018.11.018 31004884

[B58] Xiao D. Zhang Y. Wang R. Fu Y. Zhou T. Diao H. (2019b). Emodin alleviates cardiac fibrosis by suppressing activation of cardiac fibroblasts via upregulating metastasis associated protein 3. Acta Pharm. Sin. B 9 (4), 724–733. 10.1016/j.apsb.2019.04.003 31384533 PMC6664101

[B59] Xu K. Al-Ani M. K. Wang C. Qiu X. Chi Q. Zhu P. (2018a). Emodin as a selective proliferative inhibitor of vascular smooth muscle cells versus endothelial cells suppress arterial intima formation. Life Sci. 207, 9–14. 10.1016/j.lfs.2018.05.042 29803662

[B60] Xu K. Zhou T. Huang Y. Chi Q. Shi J. Zhu P. (2018b). Anthraquinone emodin inhibits tumor necrosis factor alpha-induced calcification of human aortic valve interstitial cells via the NF-κB pathway. Front. Pharmacol. 9, 1328. 10.3389/fphar.2018.01328 30510513 PMC6252319

[B61] Xu Y. Zhang S. Rong J. Lin Y. Du L. Wang Y. (2020). Sirt3 is a novel target to treat sepsis induced myocardial dysfunction by acetylated modulation of critical enzymes within cardiac tricarboxylic acid cycle. Pharmacol. Res. 159, 104887. 10.1016/j.phrs.2020.104887 32526680

[B62] Yang Y. Jiang Z. Zhuge D. (2019). Emodin attenuates lipopolysaccharide-induced injury via down-regulation of miR-223 in H9c2 cells. Int. Heart J. 60 (2), 436–443. 10.1536/ihj.18-048 30745529

[B63] Ye B. Chen X. Dai S. Han J. Liang X. Lin S. (2019). Emodin alleviates myocardial ischemia/reperfusion injury by inhibiting gasdermin D-mediated pyroptosis in cardiomyocytes. Drug Des. Devel Ther. 13, 975–990. 10.2147/dddt.S195412 PMC6438141 30988600

[B64] Zhang H. M. Wang F. Qiu Y. Ye X. Hanson P. Shen H. (2016a). Emodin inhibits coxsackievirus B3 replication via multiple signalling cascades leading to suppression of translation. Biochem. J. 473 (4), 473–485. 10.1042/bj20150419 26621875

[B65] Zhang T. Dong D. Lu D. Wang S. Wu B. (2016b). Cremophor EL-based nanoemulsion enhances transcellular permeation of emodin through glucuronidation reduction in UGT1A1-overexpressing MDCKII cells. Int. J. Pharm. 501 (1-2), 190–198. 10.1016/j.ijpharm.2016.01.067 26850314

[B66] Zhang X. Qin Q. Dai H. Cai S. Zhou C. Guan J. (2019). Emodin protects H9c2 cells from hypoxia-induced injury by up-regulating miR-138 expression. Braz J. Med. Biol. Res. 52 (3), e7994. 10.1590/1414-431x20187994 30810622 PMC6393853

[B67] Zhao X. Y. Qiao G. F. Li B. X. Chai L. M. Li Z. Lu Y. J. (2009). Hypoglycaemic and hypolipidaemic effects of emodin and its effect on L-type calcium channels in dyslipidaemic-diabetic rats. Clin. Exp. Pharmacol. Physiol. 36 (1), 29–34. 10.1111/j.1440-1681.2008.05051.x 18785977

[B68] Zheng Q. Li S. Li X. Liu R. (2021). Advances in the study of emodin: An update on pharmacological properties and mechanistic basis. Chin. Med. 16 (1), 102. 10.1186/s13020-021-00509-z 34629100 PMC8504117

[B69] Zhong Y. Ding Y. Xiao D. Hu D. Li Y. (2022). New 18β-glycyrrhetinic acid-emodin esters synthetized by a one-step innovative route, its structural characterization, and *in vivo* toxicity assessed on zebrafish models. Chem. Biodivers. 19 (4), e202100928. 10.1002/cbdv.202100928 35243763

[B70] Zhou L. Hu X. Han C. Niu X. Han L. Yu H. (2022). Comprehensive investigation on the metabolism of emodin both *in vivo* and *in vitro* . J. Pharm. Biomed. Anal. 223, 115122. 10.1016/j.jpba.2022.115122 36327583

[B71] Zhu L. Zhao J. L. Peng X. H. Wan M. H. Huang X. Tang W. F. (2014). Pharmacological study on free anthraquinones compounds in rhubarb in rats with experimental acute pancreatitis. Zhongguo Zhong Yao Za Zhi 39 (2), 304–308. 24761651

